# Characterization and Anti-Biofilm Activity of Lytic Enterococcus Phage vB_Efs8_KEN04 against Clinical Isolates of Multidrug-Resistant *Enterococcus faecalis* in Kenya

**DOI:** 10.3390/v16081275

**Published:** 2024-08-09

**Authors:** Oumarou Soro, Collins Kigen, Andrew Nyerere, Moses Gachoya, Martin Georges, Erick Odoyo, Lillian Musila

**Affiliations:** 1 Department of Molecular Biology and Biotechnology, Pan African University Institute for Basic Sciences, Technology, and Innovation, Nairobi P.O. Box 62000-00200, Kenya; oumarousoro2@gmail.com; 2 Department of Emerging Infectious Diseases, Walter Reed Army Institute of Research-Africa, Nairobi P.O. Box 606-00621, Kenya; collins.kigen@usamru-k.org (C.K.); mosesgachoya@gmail.com (M.G.); martin.gmatata@gmail.com (M.G.); erick.odoyo@usamru-k.org (E.O.); 3 Center for Microbiology Research, Kenya Medical Research Institute, Nairobi P.O. Box 54840-00200, Kenya; 4 Department of Medical Microbiology, Jomo Kenyatta University of Agriculture and Technology, Nairobi P.O. Box 62000-00200, Kenya; knyerere@jkuat.ac.ke

**Keywords:** bacteriophage, biofilm, *Enterococcus faecalis*, genome, multidrug resistance, phage therapy

## Abstract

*Enterococcus faecalis* (*E. faecalis*) is a growing cause of nosocomial and antibiotic-resistant infections. Treating drug-resistant *E. faecalis* requires novel approaches. The use of bacteriophages (phages) against multidrug-resistant (MDR) bacteria has recently garnered global attention. Biofilms play a vital role in *E. faecalis* pathogenesis as they enhance antibiotic resistance. Phages eliminate biofilms by producing lytic enzymes, including depolymerases. In this study, Enterococcus phage vB_Efs8_KEN04, isolated from a sewage treatment plant in Nairobi, Kenya, was tested against clinical strains of MDR *E. faecalis*. This phage had a broad host range against 100% (26/26) of MDR *E. faecalis* clinical isolates and cross-species activity against *Enterococcus faecium*. It was able to withstand acidic and alkaline conditions, from pH 3 to 11, as well as temperatures between −80 °C and 37 °C. It could inhibit and disrupt the biofilms of MDR *E. faecalis*. Its linear double-stranded DNA genome of 142,402 bp contains 238 coding sequences with a G + C content and coding gene density of 36.01% and 91.46%, respectively. Genomic analyses showed that phage vB_Efs8_KEN04 belongs to the genus *Kochikohdavirus* in the family *Herelleviridae.* It lacked antimicrobial resistance, virulence, and lysogeny genes, and its stability, broad host range, and cross-species lysis indicate strong potential for the treatment of Enterococcus infections.

## 1. Introduction

*Enterococcus faecalis* (*E. faecalis*) is a Gram-positive, facultative anaerobic coccus that causes difficult-to-treat infections in the nosocomial setting [1]. It is commonly found in nature and is part of the human intestinal microbiota, comprising less than 1% of the microbiome [2,3]. Early in its evolution, *E. faecalis* acquired traits that enabled it to become an effective nosocomial pathogen, resistant to several drugs and causing severe infections in humans. It causes many human infections, including bacteremia, soft tissue and wound infections, pneumonia, endocarditis, and urinary tract infections [4,5,6]. It can persist for extended periods on medical equipment, and because of its high tolerance and genetic adaptability, *E. faecalis* is a significant contaminant in the hospital environment [7]. The ability of *E. faecalis* to form biofilms is particularly concerning in clinical settings as its biofilms form on medical equipment such as catheters and prosthetic heart valves, leading to persistent infections that exhibit increased resistance to antibiotics within the biofilm structure [8]. Biofilms are organized communities of microorganisms that attach to surfaces and are embedded in self-produced extracellular polymeric substances (EPS) consisting of proteins, extracellular DNA, and polysaccharides [9]. Bacterial biofilms enhance pathogenicity; for example, they contribute significantly to persistent chronic urinary tract infections (UTIs), including recurrences and relapses [10]. Existing antibiotics have limited efficacy in eliminating biofilms and are less effective in treating the growing number of multidrug-resistant (MDR) infections [11], prompting the exploration of phage-based therapies as promising alternatives for eradicating biofilms and treating MDR pathogens. The dramatic increase in the frequency of antibiotic therapy failures due to resistance has prompted scientists to search for novel solutions.

Bacteriophages, viruses that infect bacteria, have been investigated for the development of highly effective antimicrobials with low toxicity and minor environmental impact. Bacteriophages, known for their narrow host range, are the most represented biological entities on Earth, and their number in ecosystems is estimated to exceed 10^31^ [12]. Phages can eliminate biofilms by producing enzymes that prevent biofilm formation and disrupt existing biofilms [13]. Depolymerases and lysins are bacteriophage enzymes that selectively degrade biofilms’ extracellular polymeric substance matrix components, enhancing the phages’ access to bacterial biofilm [14].

Phages with narrow host ranges are highly specific for specific bacterial strains or species. This specificity can be advantageous when precise targeting is needed, such as treating specific bacterial infections [15]. However, this makes them less valuable when targeting a wide range of bacteria, such as when treating polymicrobial infections or during the emergence of phage resistance [16]. In this regard, a phage with a broad host range is particularly advantageous because it can target more than one bacterial strain, presumably leading to fewer treatment failures [15].

Many *E. faecalis* phages have been identified to date [17,18,19,20] and have been shown to inhibit and disrupt the biofilms of their host bacteria [21,22,23]. For instance, studies have shown the ability of phage EFDG1 to reduce two-week-old biofilms of *E. faecalis* V583 [24]. Additionally, a genetically engineered orthocluster VIII phage phiEf11 reduced the established biofilm of *E. faecalis* strains JH2-2 and V583, which had formed on coverslips [25]. After 24 and 48 h of incubation, a significant, 10–100-fold decrease in viable cells was observed [26]. Phage therapy has several potential advantages over traditional antibiotics. The phages’ host specificity reduces damage to the body’s beneficial microbiota. Furthermore, since phages are part of the human microbiota and environment, phage treatment is quite safe [27]. They can replicate within the body, increasing their numbers at the site of infection, and have a rapid clearance potential [28]. A wave of successful bacteriophage therapies has recently been reported in the United States and Europe. The Food and Drug Administration (FDA) in 2016, for the first time, granted emergency approval for Tom Patterson’s phage treatment for a life-threatening, multidrug-resistant *Acinetobacter baumannii* infection after all antibiotics failed [29,30]. Additionally, the FDA approved a clinical trial of an intravenous bacteriophage treatment for patients with ventricular assist devices who have developed drug-resistant *Staphylococcus aureus* infection [29]. The study demonstrated successful outcomes in several patients and most patients tolerated the treatment well and did not have significant side effects [31,32]. Since then, several other patients were also approved for the therapy [29,30]. Some clinical trials, including the treatment of urinary tract infections, are underway, and some preliminary results are encouraging [32]. Despite the promising nature of phage therapy in the fight against antimicrobial-resistant bacteria, a few rare case studies have identified certain limitations. As an illustration, a patient suffering from a *Pseudomonas aeruginosa* multidrug-resistant prosthetic vascular graft infection was treated using a cocktail of phages (PT07, 14/01, and PNM) in combination with ceftazidime-avibactam. The outcome, nonetheless, did not meet expectations. After phage treatment and without antimicrobial therapy, a new bloodstream infection, increased biofilm production, and the emergence of phage-resistant mutants in the bacterial isolate occurred, highlighting the challenges and potential risks associated with phage therapy in complex infections [33].

This study presents the genomic characterization and antibiofilm activity of *E. faecalis* phage vB_Efs8_KEN04, isolated from community wastewater in Nairobi, Kenya. This phage exhibits a relatively broad host range against clinical MDR *E. faecalis* isolates and a potent capacity to disrupt (eliminate already formed) and inhibit (prevent biofilm initiation) *E. faecalis* biofilms under laboratory conditions. This study also evaluates the stability of lytic phage vB_Efs8_KEN04 in vitro under different temperatures and pH ranges. The discovery of phage vB_Efs8_KEN04 offers a promising phage-based therapy to effectively combat multidrug-resistant enterococcal infections and their biofilms.

## 2. Materials and Methods

### 2.1. Strains and Cultural Conditions

Archived clinical isolates of multidrug-resistant *E. faecalis* and *Enterococcus faecium* (*E. faecium*) (37 in total, 26 MDR *E. faecalis* and 11 MDR *E. faecium*) from patients in different hospitals around Kenya were obtained from an ongoing surveillance study (protocol WRAIR-2089/KEMRI-2767) in the Department of Emerging Infectious Diseases, Walter Reed Army Institute of Research-Africa (WRAIR-A) in Kenya. Bacterial identity and antimicrobial susceptibility testing profiles were first confirmed using the Vitek 2 version 9.02 automated platform (bioMérieux, Marcy-l’Étoile, France). The bacterial isolates were cultured in tryptic soy broth (Oxoid Ltd., Basingstoke, Hampshire, UK) under aerobic conditions with agitation at 37 °C and a speed of 200 rpm for phage isolation.

### 2.2. Bacteriophage Isolation, Purification, and Propagation

Raw sewage water samples were collected from a sewage treatment plant in Nairobi East. It treats domestic and industrial wastewater, handling approximately 80% of the wastewater generated in Nairobi city daily. It is, therefore, a significant source of bacteria in the environment [34]. Phage vB_Efs8_KEN04 was isolated through an enrichment method using *E. faecalis* isolate EFS8 as a host according to the method described by D’Souza et al. with slight modifications [35]. Briefly, 50 mL of environmental wastewater was centrifuged at 12,000× *g* for 10 min (Thermo Fisher Scientific, Waltham, MA, USA). A total of 8 milliliters of wastewater sterilized by filtration through a 0.22 µm membrane was mixed with 2 mL of 5× tryptic soy broth (TSB; Oxoid Ltd., Basingstoke, Hampshire, UK) and 50 µL of bacterial culture grown in 1× TSB for 16–24 h at 37 °C with agitation at 200 rpm. The mixture was incubated for 24 h at 37 °C with agitation at 200 rpm. Bacterial debris was eliminated by centrifugation, and the supernatant was filter sterilized through a 0.22 µm filter. Serial 10-fold dilutions of phage vB_Efs8_KEN04 in sodium chloride–magnesium sulfate (SM) buffer were spotted onto double-layer (0.7% top/1.5% bottom) tryptic soy agar (TSA) agar overlaid with 100 µL of a culture of *E. faecalis* isolate EFS8 in the semisolid top layer. The next day, a well-isolated phage plaque was suspended in SM buffer and filter sterilized. Phage vB_Efs8_KEN04 was purified by three rounds of single-plaque isolation through plaque assays and propagated to reach a high titer. Briefly, the following components were mixed in a 50 mL falcon tube to amplify the phage—20 mL of TSB, 10 µL of 1M CaCl_2_, 40 µL of 1M MgCl_2_, 200 µL of 10% glucose, and 400 µL of the overnight host bacteria—and then incubated at 37 °C with shaking at 200 rpm for 1−2 h to reach the mid-log phase. When the host bacterium reached the exponential growth phase, 250 µL of a single pure phage suspension was added, followed by incubation at 37 °C, 200 rpm until lysis occurred. Bacterial debris was removed by a 10 min centrifugation at 12,000× *g*, after which the supernatant was filtered through a sterile 0.22 µm filter. The filtrate was centrifuged for 16−18 h at 10,000× *g* to pellet the phages. The supernatant was discarded after centrifugation, leaving approximately 2 mL of supernatant to resuspend the pellet. The phage titers were determined using a spot assay [36].

### 2.3. Phage Stability

#### 2.3.1. Thermal Stability

Thermal stability was determined by dispensing 20 µL of the propagated phage suspension with a titer of 2 × 10^9^ PFU/mL into 0.2 mL PCR tubes and incubating at different temperatures (−80 °C, −20 °C, +4 °C, 20 °C, 22−30 °C, 37 °C, 40 °C, 45 °C, 50 °C, and 60 °C) for 1 h. After incubation, the phage lysate was diluted in SM buffer using a 10-fold serial dilution technique in 96-well round-bottom (U) microplates (Thermo Scientific, Roskilde, Denmark), and the phage titer was then evaluated using a spot assay as described elsewhere [36]. The experiment was performed in triplicate, and the phage lysate stored at +4 °C was used as the reference titer.

#### 2.3.2. pH Stability

The effect of pH 1, 3, 5, 7, 9, 11, and 13 on phage titer and viability was studied for 1 h in TSA plates using the spot test method, as described elsewhere [36]. The pH of the SM buffer was adjusted to the desired value using 1M NaOH and 1M HCl. The pH of SM was determined using a pH meter (Thermo Scientific, Roskilde, Denmark). After incubation, the phage titer was evaluated. The experiment was performed in triplicate, and the phage lysate stored at pH 7.5 was used as the reference titer.

### 2.4. Host Range Analysis

To investigate the activity of phage vB_Efs8_KEN04 against other endemic bacterial strains, its host range was determined using a spot test [36] against a panel of 37 clinical isolates of MDR enterococci, and the efficiency of plating (EOP) was determined using a double-layer agar plate method, following a previously described protocol [37,38]. The bacterial strains used for this study were associated with skin and soft tissue infection, urinary tract infection, surgical site infection, and blood infection and were widely spread across Kenyan regions. All the tested strains were cultured in broth overnight at 37 °C. Briefly, 2 µL of an individual phage stock was spotted on a TSA plate with a lawn of 100 µL of host bacteria cultured overnight in soft agar, which was examined for bacterial lysis after 18–24 h. Host range tests were performed in duplicates. A phage was termed ‘potent’ upon infecting and lysing bacterial strain in the host range panel [39]. The EOP was calculated by dividing the average plaque-forming units (PFU) of the test bacteria by the average PFU of the host bacteria. Phages were categorized as high production (EOP ≥ 0.5) when the productive infection on the test bacteria resulted in at least 50% of the PFU found for the primary host; medium production (0.1 ≤ EOP < 0.5); low production efficiency (0.001 < EOP < 0.1); inefficient (EOP ≤ 0.001), and reference (EOP = 1) [40,41].

### 2.5. Determination of Optimal Multiplicity of Infection

The optimal multiplicity of infection (MOI) of phage vB_Efs8_KEN04 was determined as previously described with slight modifications [42]. Briefly, the ratio of bacteriophage and mid-log phase bacterial culture of *E. faecalis* EFS8 (optical density, OD600 = 0.5) was adjusted to MOIs of 1, 0.1, 0.01, and 0.001. Then, the mixture was incubated for 4 h at 37 °C with shaking at 200 rpm. The samples were centrifuged at 12,000× *g* for 10 min and the supernatants filtered through a 0.22 μm filter to remove the host bacteria. Next, the phage titer was determined using a spot assay. The mixture with a ratio of phage vB_Efs8_KEN04 to *E. faecalis* EFS8 with the highest phage titer was considered the optimal multiplicity of infection. Experiments were performed in triplicates and analyzed using GraphPad Prism 8.4.0 (GraphPad Software, Inc., San Diego, CA, USA).

### 2.6. Lytic Properties of Phage vB_Efs8_KEN04

The lysis dynamics of phage vB_Efs8_KEN04 lysis against *E. faecalis* EFS8 was determined as previously described with some modifications [42]. Briefly, phages were mixed with a mid-log phase bacterial culture (OD600 = 0.5) at a MOI of 1, 0.1, 0.01, 0.001. The mixed culture was incubated at 37 °C with shaking at 200 rpm for the duration of the experiment. OD600 readings were taken every 10 min for 2 h. An *E. faecalis* EFS8 culture without phages served as a positive control. Experiments were repeated independently three times and analyzed using GraphPad Prism 8.4.0 (GraphPad Software, Inc., San Diego, CA, USA).

### 2.7. One-Step Growth Curve

One-step growth curves were determined as previously described with a slight modification [43]. Briefly, when the culture of *E. faecalis* EFS8 reached the mid-log phase (OD600 = 0.5), bacteria were harvested by centrifugation at 10,000× *g* at 4 °C for 10 min and resuspended in fresh TSB. Then, 1 mL of bacterial culture in TSB was infected with 1 mL of purified phages at a MOI of 1 and allowed to adsorb at room temperature for 10 min. After centrifugation and removal of the supernatant containing unbound phages, pellets were resuspended in 50 mL fresh TSB medium and incubated at 37 °C with shaking at 200 rpm for up to 90 min. Hundred microliter aliquots were taken every 5 min, 10-fold serially diluted, and plated using the spot assay to determine phage titers. The burst size of the phages was calculated as the ratio of the average phage titer at the plateau phase to that at the latent phase [44]. Experiments were performed three times and analyzed using GraphPad Prism 8.4.0 (GraphPad Software, Inc., San Diego, CA, USA).

### 2.8. Adsorption Assay

The adsorption rate was performed as previously described, with some modifications [45]. Briefly, the mid-log phase of the host bacterial suspension in the TSB medium was mixed with phage solution at an MOI of 1 and incubated at 37 °C. At 0, 1, 2, 3, 4, 5, 6, 7, 8, 9, 10, 15, and 20 min postinfection; then, the mixture was collected and centrifuged immediately at 12,000× *g* for 10 min. The titers of unadsorbed phages of the supernatant were determined using a spot assay.

### 2.9. Isolation of Phage-Resistant Mutants

Phage-insensitive mutants were isolated as described previously with slight modifications [46]. Briefly, 1 mL of a mid-log phase bacterial culture (OD600 = 0.5) of *E. faecalis* EFS8 was mixed with phage vB_Efs8_KEN04 at a MOI of 1 and incubated at 37 °C with shaking at 200 rpm for 90 min to allow phage infection and killing of susceptible bacteria. After incubation, the lysate was centrifuged at 12,000× *g* for 10 min to pellet phage-resistant bacteria. The supernatant containing the phage particles was discarded and the pellet resuspended in TSB, 100-fold diluted, and 50 µL inoculated onto sheep blood agar plates. After incubating for 24 h at 37 °C, the number of colonies on each plate was counted to calculate the colony-forming units. The frequency of phage-insensitive mutants was determined by dividing the remaining viable colony counts by the initial viable colony counts [37]. Bacterial culture (without phage) served as a positive control and a phage without bacteria control plate served as a negative control to ensure no contamination of the phage preparation. All experiments were conducted in triplicate. The identity of resistant mutants was confirmed using the automated Vitek 2 version 9.02 platform (bioMérieux, Marcy-l’Étoile, France) to ensure that they were *E. faecalis* isolates and not contaminants. The resistant isolates were sub-cultured and screened by spot assay to confirm that they had acquired full phage resistance with heritable phenotype.

### 2.10. Determination of the Nature of Phage Receptor

To determine the nature (protein or polysaccharide) of the phage vB_Efs8_KEN04 receptors on the bacterial surface, a mid-log phase bacterial culture of *E. faecalis* EFS8 was incubated at room temperature (22−30 °C), 60 °C, and 100 °C for 15 min. After incubation, 1 mL of bacteria boiled to denature proteins was mixed with 1 mL of the phage (with a titer of 5 *×* 10^9^ PFU/mL) and incubated at room temperature for 10 min to allow phage adsorption. Postinfection, the mixture was collected and centrifuged immediately at 12,000× *g* for 10 min. The titers of non-adsorbed phages of the supernatant were determined using a spot assay. Phages will adsorb to the boiled bacteria if the phage receptors are polysaccharides but not if they are proteins.

### 2.11. Biofilm Formation Assay

The ability of enterococci to form biofilms was assessed using a crystal violet biomass assay [47]. Briefly, the bacterial isolates were grown overnight at 37 °C, 200 rpm in tryptic soy broth (TSB). The enterococcal cultures were diluted 1:100 in fresh TSB containing 2% glucose monohydrate (Oxoid Ltd., Basingstoke, Hampshire, UK), and 100 µL of the diluted solution was dispensed into the wells of 96-well round-bottom (U) microplates (Thermo Scientific, Roskilde, Denmark) and incubated under static conditions at 37 °C, 5% CO_2_ for 72 h without changing the medium. Wells with sterile TSB containing 2% glucose were used as controls for contamination. *E. faecalis* strain ATCC 29,212 was used as a positive biofilm control [48], whereas *E. faecalis* isolate EFS4 (ST947), an in-house isolate, was used as the negative control. The experiments were performed in triplicate. After incubation, planktonic bacteria were pipetted off, the wells were washed three times with distilled water, and the plates were allowed to air-dry for 15 min. Adhesive bacteria were fixed at 60 °C for 1 h and stained with 100 µL of 1% crystal violet for 20 min. This was followed by three washes with 100 µL of sterile distilled water to remove the excess dye. The microplates were air-dried for 15 min. Then, 100 µL of 33% glacial acetic acid was added to each well, followed by pipetting to release the bound crystal violet dye from the biofilm [49]. The stained adherent cells’ optical density (OD) was quantified at 630 nm using a microtiter plate reader (BioTek Instruments, Gen5TM version 3.10, Santa Clara, CA, USA). The strains were divided into groups based on the OD values of the bacterial biofilms. Bacterial strains were classified as follows: OD values ≤ 0.0551 as non-biofilm formers, weak biofilm-producing isolates (0.0551 < OD < 0.102), moderate biofilm formers (0.102 < OD < 0.204), and those with OD > 0.204 were classified as strong biofilm-producing bacterial strains [50,51].

### 2.12. Biofilm Inhibition Assay by Phage

The anti-biofilm effect of the phage was evaluated as described by Goodarzi et al., with some modifications [21]. To investigate the inhibitory effect of phages on biofilm formation, a 3-day-old biofilm was formed in the presence of phages. Briefly, single colonies of *E. faecalis* strains were cultured in TSB at 37 °C, 200 rpm for 24 h. After incubation, the bacterial culture was diluted 1:100 in fresh TSB, supplemented with 2% glucose. The diluted bacterial culture (100 µL) was dispensed into the wells, and 2 µL of phage lysate (titer 9 × 10^9^ PFU/mL) was added. The plates were incubated under static conditions at 37 °C, 5% CO_2_ for 72 h without changing the medium. Wells with sterile TSB containing 2% glucose were used as controls for contamination. *E. faecalis* strain ATCC 29,212 was used as a positive biofilm control, whereas *E. faecalis* isolate EFS4 (ST947) was used as the negative control. Biofilm formation was performed in triplicate for treated and untreated samples. After incubation for 72 h, the suspension was drained from the wells and rinsed with sterile distilled water three times, and biofilm fixation, staining, and OD measurements were performed as described in Section 2.11.

### 2.13. Biofilm Disruption Assay by Phage

A 2-day-old biofilm was formed in the absence of phages and then treated. Briefly, single colonies of *E. faecalis* strains were cultured in TSB at 37 °C, 200 rpm for 24 h. After incubation, the bacterial culture was diluted 1:100 in fresh TSB, supplemented with 2% glucose. The diluted bacterial culture (100 µL) was dispensed into the wells, followed by incubation under static conditions at 37 °C, 5% CO_2_ for 48 h without changing the media. After 48 h of incubation, the plates were removed from the incubator, the planktonic bacteria were pipetted off, the wells were washed twice to remove all planktonic cells, and 100 µL of the phage lysate with a titer of 2 × 10^9^ PFU/mL was added. For untreated wells, the medium was replaced with TSB supplemented with 2% glucose; for treated wells, phage lysate was first mixed with TSB supplemented with 2% glucose and this mixture was used for the treatment. The plates were then placed back in the incubator for 24 h. Biofilm formation was performed in triplicate for treated and untreated samples. After 24 h of treatment, the suspension was drained from the wells and rinsed three times with sterile distilled water. Biofilm fixation, staining, and OD measurements were performed as described in Section 2.11.

### 2.14. Genomic DNA Extraction

Before DNA extraction, the pure phage suspension (2 × 10^9^ PFU/mL) was propagated to reach a titer of 3.5 × 10^11^ PFU/mL, as described in Section 2.2. One milliliter of the propagated phage suspension was treated with 0.07 mg/mL of RNase A (Thermo Fisher Scientific, USA) and 2.5 U/mL of DNase I (ThermoFisher Scientific, Waltham, MA, USA) to remove host RNA and DNA, respectively. Deproteinization was achieved by adding proteinase K and incubating at 56 °C for 1 h 30 min [35]. Phage DNA was isolated and purified using the Norgen phage DNA isolation kit (Norgen Biotek Corporation, Thorold, ON, Canada), following the manufacturer’s instructions. The quality and quantity of the extracted DNA were determined using a Nanodrop One spectrophotometer and a Qubit4 fluorometer (Fisher Scientific, Waltham, MA, USA), respectively.

### 2.15. Genome Sequencing and Bioinformatic Analysis of Sequencing Data

The genomic library was prepared using an Illumina Collibri^TM^ PCR-free ES DNA library preparation kit (Illumina, San Diego, CA, USA) and purified using the Collibri^TM^ DNA library cleanup kit (Illumina, San Diego, CA, USA). The genome was sequenced on the Illumina MiSeq platform (Illumina, San Diego, CA, USA). The quality of the raw reads was assessed using FastQC v0.12.1 [52], trimmed with fastp v0.23.4 [53], and assembled using shovill v1.1.0 (https://github.com/tseemann/shovill, accessed on 27 November 2023). Genome annotation was performed using pharokka 1.5.1 [54]. The complete phage genome was further queried against CRISPR-Cas Finder (https://proksee.ca/, accessed on 11 December 2023), PhageLeads [55] (https://phageleads.dk/, accessed on 11 December 2023), PhageTerm 1.0.11 platforms [56] (https://cpt.tamu.edu/galaxy-pub, accessed on 27 November 2023) to determine CRISPR-like systems, lysogeny genes, and termini in the phage genome, respectively. ARAGORN v1.2.41 [57] and tRNAscan-SE v2.0.12 [58] were used to predict the tRNA and tmRNA genes. Nucleic acid sequence similarity searches were performed using default parameters in BLASTn [59]. The identification of antimicrobial resistance and virulence genes was conducted by scanning the assembled nucleotide sequence using Abricate version 1.0.1 [60] at https://github.com/tseemann/abricate, accessed on 27 November 2023, with the following datasets: NCBI AMRFinderPlus [61], Comprehensive Antibiotic Resistance Database (CARD) [62], Virulence FactorDatabase (VFDB) [63], and ResFinder [64].

### 2.16. Phylogenetic Tree and Comparative Genomics of Phage Genomes

To examine the genetic relationships between Enterococcus phage vB_Efs8_KEN04 and other Enterococcus phages, a phylogenetic tree was generated using the entire genome sequences of 38 phages retrieved from the NCBI database (https://blast.ncbi.nlm.nih.gov/Blast.cgi, accessed on 23 December 2023), including phage vB_Efs8_KEN04. The phages included in the phylogenetic tree were selected according to the following criteria: (i) phages should have a complete genome sequence [65]; (ii) they should exhibit a high similarity of >70% to phage vB_Efs8_KEN04 [66]; and (iii) they should have a genome size similar to that of phage vB_Efs8_KEN04 [67]. The analysis was conducted using the Virus Classification and Tree Building Online Resource (VICTOR), a method for the whole genome-based phylogeny that makes use of the Genome BLAST Distance Phylogeny (GBDP) method, which calculates accurate intergenomic distances between pairs of viruses [65] (https://victor.dsmz.de, accessed on 23 December 2023), enabling the evaluation of similarities and differences in genetic characteristics. The nucleotide sequences were compared using the genome explosion distance phylogeny (GBDP) method in the settings recommended for prokaryotic viruses [68], and the branch length was magnified using the distance formula d0, according to GBDP. The formula d0 is used when nucleotide sequences of prokaryotic viruses are analyzed [65] and it corresponds to the length of all high-scoring segment pairs divided by the total genome length (https://ggdc.dsmz.de/faq.php#qvictor7, accessed on 10 December 2023). In addition, the intergenomic similarities between phage vB_Efs8_KEN04 and the 20 closest related Enterococcus phages were determined using a virus intergenomic distance calculator (VIRIDIC) [69] to further our understanding of their interactions. The nucleotide identity of the complete genome length cut-off for genera (>70%) and species (>95%) was used [66].

### 2.17. Phage Host Range Prediction Based on Phage Receptor-Binding Proteins

To predict the host range of phage vB_Efs8_KEN04, protein sequence similarity searches were performed with the web-based proteinBLAST tool (https://blast.ncbi.nlm.nih.gov/Blast.cgi, accessed on 20 June 2024) using tail fiber protein sequences (CDS143) of phage vB_Efs8_KEN04 obtained after genome annotation. From the blastp results, sequences with annotations unrelated to receptor-binding proteins (RBPs) (such as hypothetical proteins, structural components of the tail fiber, glycerophosphoryl diester phosphodiesterase, and tail proteins) were discarded. Only significant hits for annotated tail fiber proteins with an E-value of 0, percent identity > 95%, and protein length similar to the length of the phage vB_Efs8_KEN04 tail fiber protein were retained [70]. Information on each annotated RBP (tail fiber protein) was collected, including the protein name, the organism (phage) name, the phage host name, the percent identity, the protein length, and the protein accession number. If the phage host strain was not provided, it was determined through the literature review based on published papers.

### 2.18. Statistical Analysis

Statistical analysis of the biofilm results was conducted using GraphPad Prism 8.4.0 (GraphPad Software, Inc., San Diego, CA, USA), and a Student’s *t*-test was employed to determine significance. Statistical significance was set at *p* < 0.05.

## 3. Results

### 3.1. Bacteriophage Isolation and Purification

**Figure 1:**
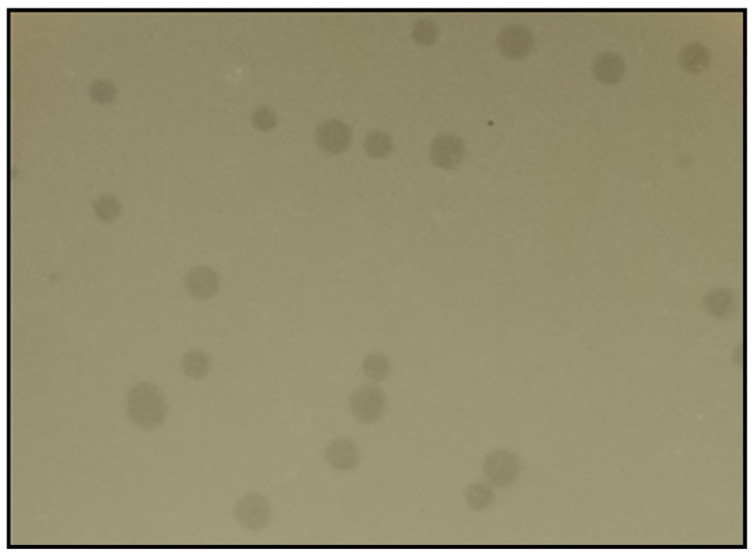
Plaque morphology of phage vB_Efs8_KEN04.

Phage vB_Efs8_KEN04 was isolated from a wastewater plant using the *E. faecalis* isolate EFS8 as the host strain. Phage vB_Efs8_KEN04 forms clear plaques on a double-layered agar plate, as shown in Figure 1.

### 3.2. Host Range Analysis

The host range and efficiency of plating studies were conducted on 37 MDR Enterococci isolates, with 26 being *E. faecalis* isolates and the remaining 11 being *E. faecium* isolates. These bacteria were isolated from urinary tract infections, skin and soft tissue infections, surgical site infections, and blood infections in humans. *E. faecalis* phage vB_Efs8_KEN04 was active against all 26 MDR *E. faecalis* and 1/11 of the *E. faecium* in the spot assay (Table 1). The EOP was greater than 0.5 for 13/26 (50%) *E. faecalis* isolates, indicating high production of the phage, and an EOP ≥ 0.5 is considered good for therapy [41].

### 3.3. Host Range Prediction Based on Receptor-Binding Proteins

The majority of phages use their RBPs (tail fiber proteins or tailspike proteins) located at the extremity of their tail to recognize and attach to specific receptors on the surface of their hosts [71,72]. If a phage has RBPs similar to those of phages that infect their bacterial host species, it is possible that the phage also infects these host strains using similar receptors on the bacterial surface to facilitate infection. In order to determine potential hosts of phage vB_Efs8_KEN04, similarity searches were performed on Blastp using the annotated tail fiber protein sequences of phage vB_Efs8_KEN04 and the results are shown in Table 2. The BLASTp results revealed high similarities (>95%) between the tail fiber protein of phage vB_Efs8_KEN04 and those of eleven other similar Enterococcus phages infecting both *E. faecalis* and *E. faecium* strains, as listed in Table 2. This indicates that phage vB_Efs8_KEN04 could potentially infect these Enterococcus strains, which include vancomycin-resistant *E. faecium* strains (VRE001, VRE004, VRE008, VRE1147, VRE1181) and the *E. faecalis* phage-resistant strain EFDG1r. However, experimental validation in the laboratory is essential to confirm this predicted host range.

### 3.4. Phage Stability

**Figure 2:**
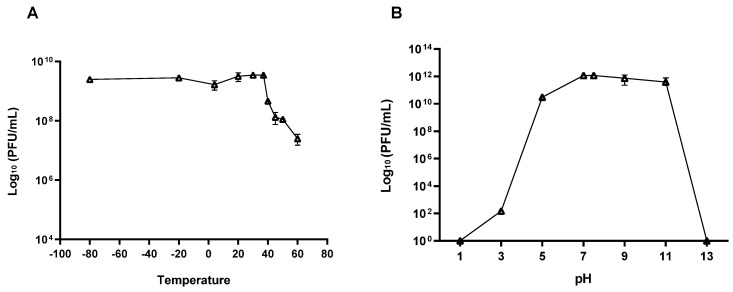
Phage stability test of Enterococcus phage vB_Efs8_KEN04. (**A**) Thermal stability test. (**B**) pH stability test. Experiments were performed in triplicate. The triangle symbols represent individual data points and the error bars represent the standard deviation.

The stability of phage vB_Efs8_KEN04 was evaluated at different temperatures and pH values. The results revealed that phage vB_Efs8_KEN04 was stable from −80 °C to 37 °C (Figure 2A and Table S1). Phage titer declined at temperatures of 40 °C and above. Similarly, the stability rate of phage vB_Efs8_KEN04 was high at pH 5–11 (slightly acidic to strongly basic) but low at pH 3 (strongly acidic). No phage activity was observed at pH 1 or 13 (Figure 2B and Table S2). These findings suggest that phage vB_Efs8_KEN04 can withstand moderate acidic and alkaline conditions and a wide range of temperature conditions between −80 °C and 37 °C.

### 3.5. Lytic Characteristics of Phage vB_Efs8_KEN04

**Figure 3:**
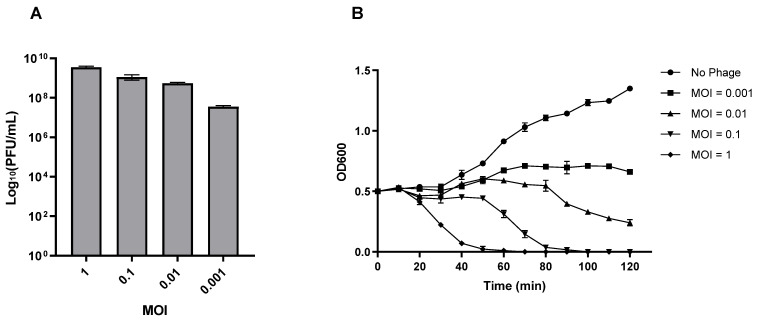
Lytic characteristics of phage vB_Efs8_KEN04. (**A**) Phage yield using different multiplicity of infections (MOIs) to determine the optimal MOI. (**B**) Phage vB_Efs8_KEN04 lysis dynamics against *E. faecalis* EFS8.

The optimal multiplicity of infection of the phage vB_Efs8_KEN04 was 1 (Figure 3A). Lower starting MOIs resulted in decreasing total phage titer. The dynamics of host strain lysis were measured at various MOIs of phage vB_Efs8_KEN04. The most rapid and robust lytic effect was demonstrated with an MOI of 1 starting after 20 min incubation and reaching full lysis at 60 min (Figure 3B). Reduced MOIs resulted in increasing delay in and completeness of observed lysis.

### 3.6. Adsorption Efficiency and One-Step Growth Curve

**Figure 4:**
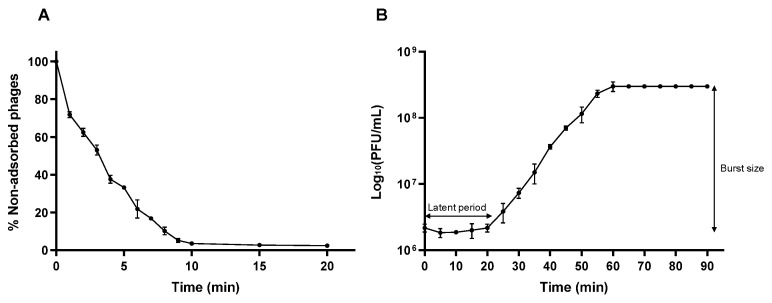
(**A**) Adsorption kinetics of the phage to its host. (**B**) One-step growth curve of phage vB_Efs8_KEN04 at a MOI of 1. Experiments were performed in triplicate, and the error bars represent the standard deviation.

To determine the adsorption rate of phage vB_Efs8_KEN04 on the surface of *E. faecalis* EFS8, an adsorption assay was performed (Figure 4A) and the percentage of non-adsorbed phages was determined. The data revealed that around 67% of the phage rapidly attached to the *E. faecalis* EFS8 within 5 min, and 97% within 10 min. In addition, a one-step growth curve showed that the latent period of phage vB_Efs8_KEN04 was approximately 20 min, followed by a rise phase of 40 min, and a plateau phase of 60 min after the initial infection (Figure 4B). The average burst size was estimated to be 138.46 ± 18.45 plaque-forming units per infected cell.

### 3.7. Phage Mutation Rate and Nature of Phage Receptors

Bacteria can protect themselves from phage infection using a range of mechanisms, such as blocking phage entry, utilizing restriction-modification systems, initiating abortive infections, and deploying CRISPR-Cas systems [81,82,83]. To determine the level of bacterial resistance to the phage, we estimated the phage mutation frequency. The mutation rate of the phage vB_Efs8_KEN04 mutants was calculated as 1.8 × 10^−5^.

**Figure 5:**
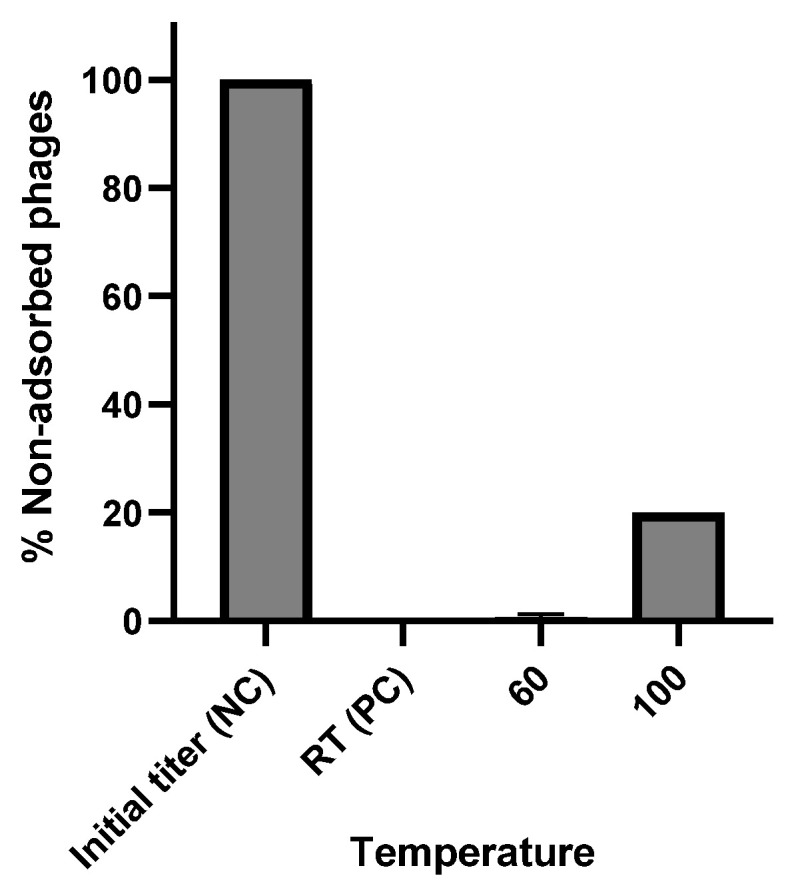
Determination of phage vB_Efs8_KEN04 receptor nature through adsorption rate measurements at different incubation temperatures. RT: room temperature (22−30 °C), NC: negative control, PC: positive control. Experiments were performed in triplicate, and the error bars represent the standard deviation.

To determine the nature of the phage receptors, the bacteria were denatured by high temperature and adsorption rates were measured. At 60 °C, almost all phages were adsorbed and the adsorption rate was similar to that of the native bacteria of the positive control. However, when the bacteria were boiled at 100 °C, ~80% of the phages were adsorbed (Figure 5). These findings indicate that the receptors are carbohydrate-based as bacterial surface proteins typically denature at a temperature of 60 °C and above, while polysaccharides are more heat-resistant. The reduction in phage adsorption rate at 100 °C suggests that even bacterial surface polysaccharides begin to degrade at this high temperature [84].

### 3.8. Biofilm Formation of Enterococcus faecalis

**Figure 6:**
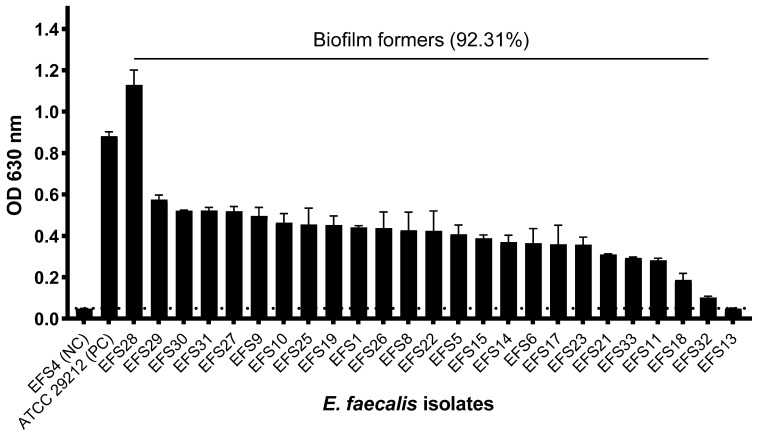
The biofilm formation profile of *Enterococcus faecalis* isolates. The biofilm formation experiment was performed in triplicate, and the error bars represent the standard deviation. The horizontal grid line represents the *E. faecalis* isolates that met the OD threshold of 0.0551 for biofilm-forming isolates.

Of the 26 clinical MDR *E. faecalis* isolates that were examined for biofilm formation, 22 isolates (84.62%) were strong biofilm formers, one isolate (3.85%) was identified as a weak biofilm former, one (3.85%) isolate as a moderate biofilm former, and two isolates (7.69%) as non-biofilm formers (Table S3). A total of 24 MDR *E. faecalis* isolates (92.31%) showed the ability to produce biofilms. *E. faecalis* isolate EFS8, the phage host, was a strong biofilm former (Figure 6).

### 3.9. Biofilm Inhibition and Disruption by Phage vB_Efs8_KEN04

**Figure 7:**
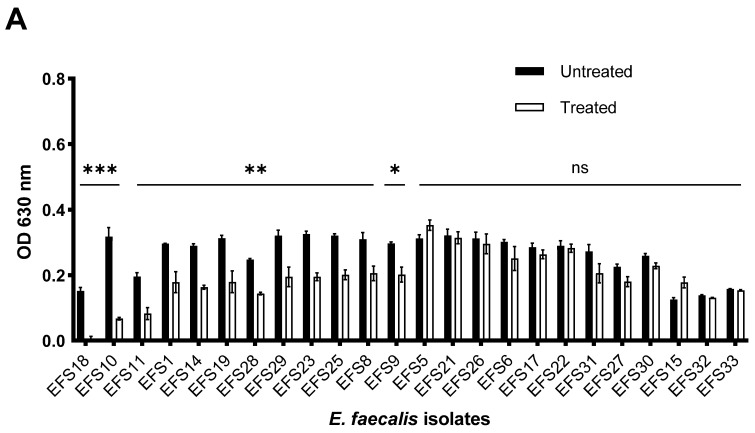

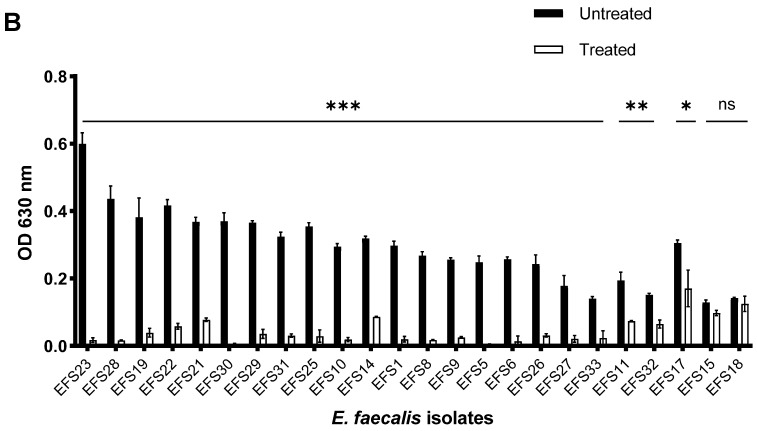
(**A**) Inhibition of biofilm by Enterococcus phage vB_Efs8_KEN04. (**B**) Biofilm disruption by phage vB_Efs8_KEN04. Biofilm inhibition and disruption experiments were performed in triplicate, and the error bars represent the standard deviation. Significance level: *, *p* < 0.05 significant; **, *p* < 0.01 very significant; ***, *p* < 0.001 highly significant; ns, not statistically significant.

The effects of phage vB_Efs8_KEN04 treatment on the inhibition and disruption of biofilms of multidrug-resistant *E. faecalis* isolates are shown in Figure 7. For the inhibition of biofilm formation (Figure 7A and Table S4), phage vB_Efs8_KEN04 treatment for 72 h at 37 °C reduced the bacterial population significantly (**, *p* < 0.05) for some susceptible bacteria. However, it could not prevent other bacteria (EFS5, EFS6, EFS15, EFS17, EFS21, EFS22, EFS26, EFS27, EFS30, EFS31, EFS31, EFS32, and EFS33) from forming biofilms (*p* > 0.05, highlighted by ns). Already formed biofilms were treated with phage vB_Efs8_KEN04 for 24 h. It significantly disrupted biofilms and reduced the bacterial population (**, *p* < 0.05) (Figure 7B and Table S5) for all the bacteria, including its host bacteria EFS8, except for the isolate EFS18 (ns, *p* > 0.05).

### 3.10. Genome Characteristics of Enterococcus faecalis Phage vB_Efs8_KEN04

**Figure 8:**
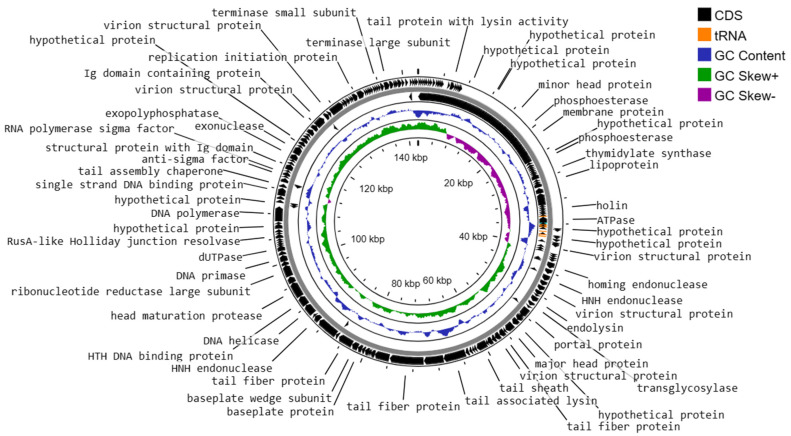
Circular genome map of Enterococcus phage vB_Efs8_KEN04 constructed using CGView.

The genome structure of phage vB_Efs8_KEN04, a newly isolated *E. faecalis* phage in Kenya, was investigated in this study. The phage genome contained 8 tRNA genes and was shown to be a linear double-stranded DNA with a length of 142,402 base pairs and a G + C content of 36.01% (Figure 8). It belongs to the genus *Kochikohdavirus* of the family *Herelleviridae*. The genome contained 238 coding sequences (CDS) with a coding gene density of 91.46%. A total of 70 CDSs (29.41%) were predicted to encode functional proteins, and the remaining 168 (70.59%) were annotated as hypothetical proteins (Table S6). The functional proteins were divided into the following categories:

(i) DNA replication, transcription, translation, and nucleotide metabolism: A total of 25 CDSs were predicted to encode for DNA replication, transcription regulation, translation, and metabolism-related proteins, such as HNH homing endonuclease, DNA helicase, exonucleases, transcriptional repressor, DNA helicase, DNA primase, and a transcriptional regulator, RNA polymerase beta subunit, and thymidylate synthase.
(ii) Structural and packaging proteins: 27 CDS were predicted to encode for tail, head, and packaging proteins such as portal proteins, head proteins, tail fiber proteins, head maturation proteases, virion structural proteins, tail proteins, tail assembly chaperones, minor and major head proteins, and terminase large and small subunits.
(iii) Host lysis and adhesion proteins: Two CDS were predicted to encode holin and endolysin proteins. BLASTp analysis of the phage vB_Efs8_KEN04 genome revealed no similarities to the genes encoding integrase or excisionase. The genome of phage vB_Efs8_KEN04 lacks genes encoding toxins, virulence factors, antibiotic resistance genes, and CRISPR. These data indicate that phage vB_Efs8_KEN04 is a strictly lytic phage that can be used to treat *E. faecalis* infection.
(iv) Sixteen CDS encode for moron, auxiliary metabolic genes, and host takeover.

Analysis of phage vB_Efs8_KEN04 DNA termini and phage packaging mechanisms revealed long direct terminal repeats (DTR) of 2849 bp with a specific packaging site called the cos site, which serves as a recognition signal for the packaging machinery. This DTR is comparable to that of bacteriophage T5 [85].

### 3.11. Phylogenetic Analysis

To gain a deeper understanding of the evolution and relationship between phage vB_Efs8_KEN04 and other Enterococcus phages, the genome of phage vB_Efs8_KEN04 was compared with that of 37 Enterococcus phages. These phage sequences were obtained from the National Center for Biotechnology Information (NCBI) database, and all had identities ranging from 78.06 to 99.29% with phage vB_Efs8_KEN04 (Table S7).

**Figure 9:**
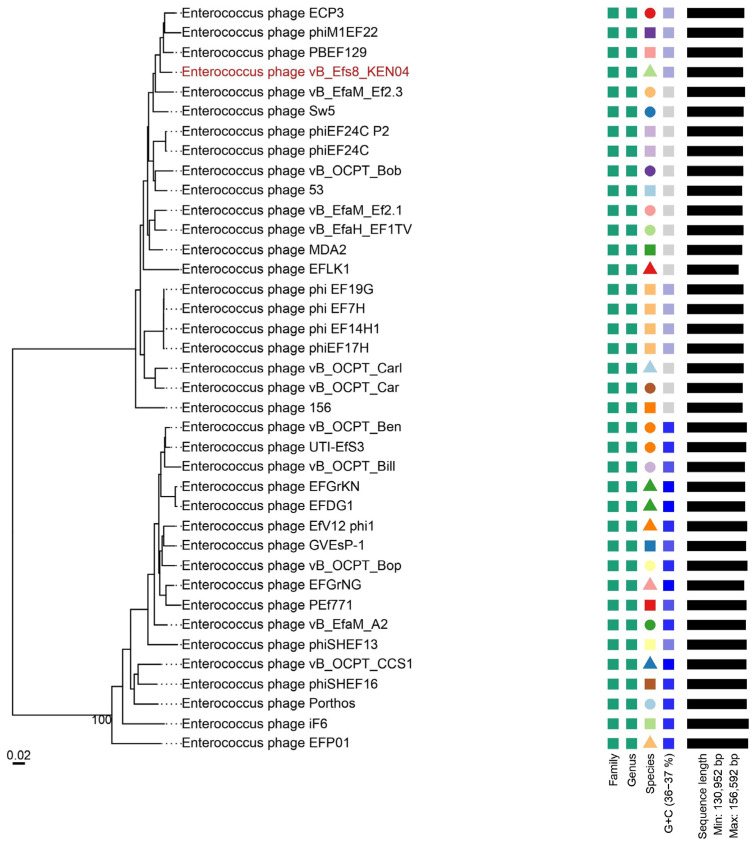
Phylogenetic analysis of Enterococcus phage vB_Efs8_KEN04 and other related Enterococcus bacteriophages based on the similarity of whole genome sequences. The phylogenetic tree was generated using the online Virus Classification and Tree Building Online Resource (VICTOR) platform with the formula d0 [65]. Color code legend from left to right: green squares in the first column correspond to the phages’ family cluster; green squares in the second column correspond to the phages’ genus cluster; the third column corresponds to the phages’ species cluster; the fourth column corresponds to the GC content (36-37%); and the fifth column corresponds to the genome length (min: 130,952 bp; max: 156,952 bp). The first and second column color codes and shapes indicate that all the phages are classified in the same family and genus, respectively. In the third and fourth columns, if the shape, color, and color intensity are the same, it means that the phages shared the same characteristics (species or GC content) but if they differed, it means that the phages are different species or have different GC contents.

**Figure 10:**
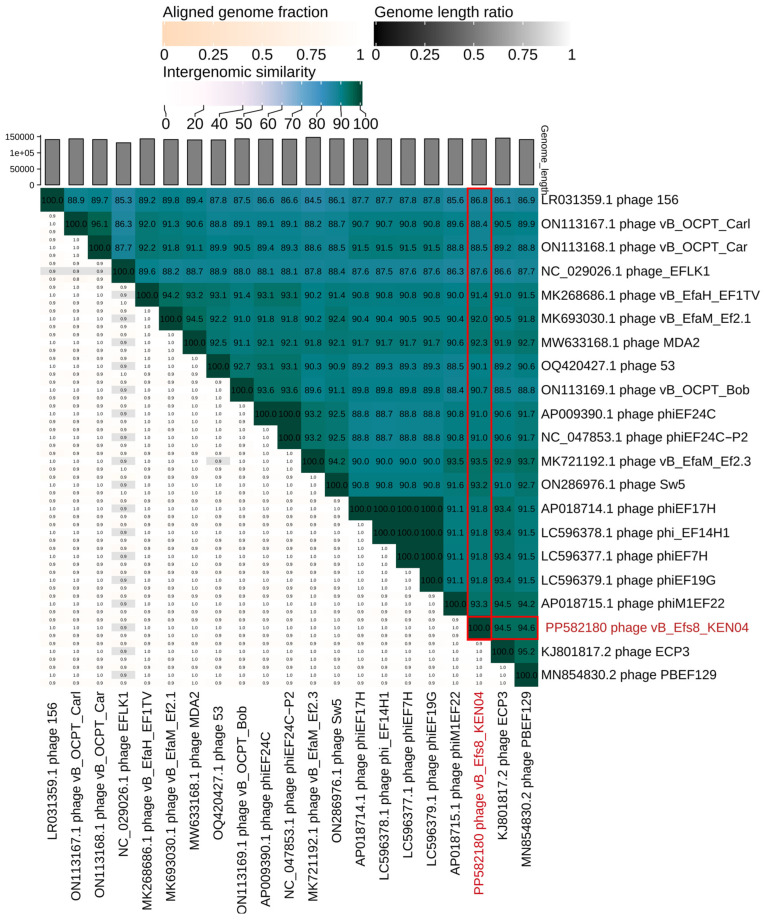
Heatmap of the average nucleotide identity values between phage vB_Efs8_KEN04 and the top 20 most similar Enterococcus bacteriophages. In the right half, the numbers represent the similarity values for each genome pair. In the left half, three indicator values are presented for each genome pair, in the order from top to bottom: aligned fraction genome 1 (for the genome found in this row), genome length ratio (for the two genomes in this pair), and aligned fraction genome 2 (for the genome found in this column). The vertical and horizontal red boxes indicate the intergenomic similarities between phage vB_Efs8_KEN04 and the most similar Enterococcus phages. The vertical and horizontal red text refer to the phage vB_Efs8_KEN04 isolated in this study and its GenBank accession number.

The phylogenetic tree, generated with the whole genome sequences, indicated that phage vB_Efs8_KEN04 had the highest similarity to Enterococcus phage PBEF129 (GenBank accession number MN854830.2), Enterococcus phage phiM1EF22 (GenBank accession number AP018715.1), Enterococcus phage ECP3 (GenBank accession number NC_027335.2), and Enterococcus phage vB_EfaM_Ef2.3 (GenBank accession number MK721192.1) (Figure 9 and Table S7). Subsequently, we employed VIRIDIC to compute the intergenomic similarities, revealing the degree of similarity between phage vB_Efs8_KEN04 and the top 20 phages most closely linked to it (Figure 10). This indicated that the similarity of phage vB_Efs8_KEN04 and the other Enterococcus phages’ complete genome was significantly greater than the genus threshold of 70% and lower than the species threshold of 95% [66], suggesting that they belong to the same genus but distinct species. The intergenomic similarities between phage vB_Efs8_KEN04 and the four most closely related Enterococcus phages were as follows: Enterococcus phage PBEF129 (94.6%), Enterococcus phage phiM1EF22 (93.3%), Enterococcus phage ECP3 (94.5%), and Enterococcus phage vB_EfaM_Ef2.3 (93.5%) (Figure 10).

## 4. Discussion

The use of bacteriophages in treating bacterial infections, including *E. faecalis*, has, in recent years, gained significant attention. This is due to the increase in antibiotic resistance and the phages’ ability to infect and eliminate bacteria. Phages have the potential to serve as a natural, safe, and efficient method for preventing and controlling multidrug-resistant (MDR) organisms [86]. Studies have also shown that phages can be used to control biofilms formed by *E. faecalis* [23,87].

This study characterized a highly lytic and broad-spectrum *E. faecalis* phage vB_Efs8_KEN04 isolated from environmental wastewater in Nairobi, Kenya. It is a dsDNA phage belonging to group I in Baltimore’s classification of viruses [88]. The genome of this phage did not encode any lysogenic, antibiotic resistance, or virulence and CRISPR-cas genes. Thus, it is an effective and safe candidate for phage therapy. Based on genome annotation, phage vB_Efs8_KEN04 was classified as a member of the genus *Kochikohdavirus* in the family *Herelleviridae*. Its genome contains eight genes encoding for transfer RNAs (tRNAs). The presence of tRNAs in bacteriophage genomes is widespread, especially among virulent phages [89]. However, their precise role has remained ambiguous for almost five decades as phages utilize the host’s transcriptional machinery to control the expression of their own genes after the initial infection [90]. Several hypotheses have been proposed for the role of these phage-encoded tRNAs. The most established is codon compensation where codons, rarely used by the host but necessary to the phage, are supplemented by the tRNAs encoded by the phage [89]. Recently, a study proposed a new hypothesis that phage-encoded tRNAs counteract the tRNA-depleting strategies of the host using enzymes such as VapC, PrrC, Colicin D, and Colicin E5 to defend against a viral infection, and they have evolved to be insensitive to host anticodon nucleases [91].

Phage vB_Efs8_KEN04 has an optimal MOI of 1 and a high adsorption efficiency, achieving over 97% attachment within 10 min. Additionally, it has a short latent period of 20 min and a relatively high burst size of 138.46 ± 18.45 PFU per bacterium, in contrast to most tail phages, which have a median latency period and burst size of 40–60 min and 50–100, respectively [77]. It also has an EOP ≥ 0.5 for 50% (13/26) of *E. faecalis* isolates, indicating high progeny production per infected cell. The observed burst size was considered large in comparison to other *E. faecalis* phages, which have reported average burst sizes of 5.7 PFU/cell for phage SAM-E.f 12 [92], 40 PFU/cell for phage LG1 [17], 70 PFU/cell for phage vB_EfaS-271 [93], and 83 PFU/cell for phage PBEF129 [94]. However, larger burst sizes have been reported for Enterococcus phages HEf13 and PEF7b (352 PFU/cell) [19,43], PEF9 (303 PFU/cell), PEF1 (262 PFU/cell) [19], and EfKS5 (183.33 PFU/cell) [22]. Variations in the latent period and burst size of phages can be attributed to the type of host cells, growth medium, pH, and temperature of incubation [95]. Phages with a large burst size are considered more virulent as they can rapidly and effectively eliminate bacterial infections [96].

Phage vB_Efs8_KEN04 exhibited a wide host range by displaying lysis activity against all 26 clinical MDR *E. faecalis* isolates tested. These isolates belong to various sequence types (ST6, ST16, ST28, ST44, ST59, ST368, ST947, ST1903, ST1904, ST1907, and ST1908) and are associated with different infection types, such as urinary tract infection, skin and soft tissue infection, surgical site infection, and blood infection. Enterococcus phages with broad host ranges have previously been reported. For example, *E. faecalis* phages EF17H and EF24C were shown to have broad host ranges, infecting 91% and 89% of tested hosts, respectively, irrespective of the bacterial clinical origin [77]. In addition, phage vB_Efs8_KEN04 showed cross-species activity against a clinical isolate of multidrug-resistant *E. faecium*. This lytic effect of *E. faecalis* phages toward *E. faecium* has been reported on the *E. faecalis* phage Max [97], phage EFRM31 [98], and the siphovirus IME-EF1 [20]. The phage’s ability to target its host bacteria is due to its host receptors involved in recognition, interaction, and adsorption during the phage attachment [99]. Additionally, the receptors are recognized by the ends of the virion’s long tail fibers of the phage toward the host bacteria [100]. The BLASTp similarity searches revealed that the tail fiber protein of phage vB_Efs8_KEN04, showed high similarity to the tail fiber proteins of eleven other similar Enterococuss phages, including phages MDA2, phiM1EF22, ECP3, phiEF17H, vB_OCPT_Carl, EF24C, and vB_OCPT_Bob, with percent identity ranging from 95.99% to 99.95%. This similarity in RBPs suggests that phage vB_Efs8_KEN04 might recognize and bind to the same or similar receptors on the host bacteria of these closely related phages, facilitating its infection. Based on its tail fiber protein similarities, phage vB_Efs8_KEN04 is predicted to potentially infect vancomycin-resistant *E. faecium* strains (VRE001, VRE004, VRE008, VRE1147, VRE1181), which are among the host range of phage MDA2 [73]. Additionally, it could infect several *E. faecalis* strains, including vancomycin-resistant strains EF09PII, EF116PII, V587 [76], biofilm-forming *E. faecalis* V583, and the *E. faecalis* V583 phage-resistant mutant EFDG1r, which developed resistance to Enterococcus phage EFDG1 [24,80].

The nature and locations of host cell receptors recognized by phages differ widely based on the specific phage and host. These receptors can include a variety of structures, from peptide sequences to polysaccharide moieties and can be located in the bacterial cell walls, capsules, or slime layers [99]. This study revealed that phage vB_Efs8_KEN04 receptors are carbohydrate-based. However, additional experiments are essential to identify the exact type of carbohydrate and the location of the receptors on the bacterial surface. The current knowledge on *E. faecalis* phage receptors, the molecular basis of phage strain specificity, and the mechanisms by which *E. faecalis* develops phage resistance is limited. However, one study has reported that the *E. faecalis* phage efap05-1 may encode various receptor-binding proteins, enabling it to adsorb to both polysaccharides and membrane proteins [101]. Another study found that phages VPE25 and VFW require PIP_EF_, an integral membrane protein, for their adsorption [102]. Identifying phage receptors is crucial for the rational selection of phages for therapeutic purposes and for understanding the mechanisms of phage resistance [103]. In this study, we determined that *E. faecalis* EFS8 can develop resistance to phage vB_Efs8_KEN04 with a mutation frequency of 1.8 × 10^−5^. Host bacteria have developed a multitude of sophisticated and complex mechanisms to escape phage infection. These include preventing phage adsorption through receptor modification or blocking [104], escaping phage infection by employing superinfection exclusion systems, restriction-modification systems, abortive infection systems, CRISPR-Cas adaptive immunity, toxin-antitoxin systems, and phage-induced chromosome islands [104,105,106,107]. Further research on phage-host interactions is essential to elucidate the resistance mechanisms of *E. faecalis* EFS8 to phage vB_Efs8_KEN04.

When subjected to different temperatures and pH conditions, phage vB_Efs8_KEN04 showed the ability to withstand moderate acidic and alkaline conditions from pH 3–11 and a wide temperature range from −80 °C to 37 °C. Many external physical and chemical factors, including but not limited to temperature, acidity, salinity, and ions, determine bacteriophage occurrence, viability, and storage. These factors can inactivate the phage by damaging its structural components (head, tail, envelope), lipid depletion, and/or DNA structural changes [108]. The studied phage exhibited remarkable stability throughout a broad range of temperatures and pH levels, making it advantageous for formulation into a suitable pharmaceutical form and therapeutic applications. Furthermore, the phage’s stability across acidic and alkaline environments (pH 3–11) enables it to be administered orally without compromising its viability in the gastrointestinal tract [23].

In this study, we also investigated the effect of phage vB_Efs8_KEN04 on the biomass reduction of *E. faecalis* biofilm by inhibition and disruption experiments. Biofilms are communities of bacteria that can be highly resistant to antibiotics and contribute to persistent infections [24,109]. Several factors contribute to the enhanced antimicrobial resistance of microorganisms in a biofilm. These include the physical barrier created by the extracellular matrix, which hinders the diffusion of antimicrobial agents [110]. Additionally, nutrient and oxygen depletion within the biofilm can cause bacteria to enter a stationary state, making them less susceptible to microbial killing [111].

Furthermore, a subpopulation of bacteria might differentiate into a phenotypically resistant state, and some bacteria within the biofilm have been found to express specific antimicrobial resistance genes unique to biofilms [112]. Recent studies have demonstrated that extracellular DNA (eDNA) in the biofilm matrix protects microbial cells against various antimicrobial agents [113]. The biofilm formation phenotype of multidrug-resistant *E. faecalis* was investigated, and the results revealed that out of 26 MDR tested for this purpose, 92.31% showed the ability to form biofilms. To date, several investigations have been performed to test bacteriophages’ ability to inhibit and destroy *E. faecalis* biofilms [17,22,87,103], but the mechanisms of the phage–biofilm interaction are not well understood [114]. As indicated by the crystal violet biomass assay, the isolated phage vB_Efs8_KEN04 significantly reduced biofilm biomass (*p*-value < 0.05) compared with the control for most of the biofilm-forming *E. faecalis* isolates. Based on genome and structural proteome analysis, this can be explained by the production of depolymerases, such as endolysins [115] (CDS121) (Table S6), which penetrate the inner layers of the biofilm by degrading structural components of the established biofilm exopolymeric matrix, allowing them to break it down or disrupt its integrity, [116] and lyse bacteria at the edge of the EPSs [117]. The reduction of bacteria on the biofilm causes the reduction of EPS material; thus, the biofilm is completely eliminated [118].

Similarly, phage vB_Efs8_KEN04 was able to significantly decrease biofilm biomass when compared with an already formed untreated biofilm. Therefore, phage vB_Efs8_KEN04 has the potential to be successfully used as a biofilm eradication agent. The phage could not inhibit the biofilm formation of several isolates (EFS5, EFS6, EFS15, EFS17, EFS21, 324 EFS22, EFS26, EFS27, EFS30, EFS31, EFS31, EFS32, EFS33) and could not disrupt already formed biofilms of two isolates (EFS15, EFS18). This can be due to factors such as high-density biofilm, sub-populate phage resistance in biofilm, and inhibition of phage infection via quorum sensing [119]. To address this limitation of phage effectiveness and eradicate bacterial biofilms more efficiently, the use of bacteriophage cocktails, containing two or more phages, with different host ranges and modes of action is an alternative, as phage cocktails can prevent further accumulation and diffusion of biofilms by reducing migratory bacteria, increase activity by expanding the host range, and prevent the formation of phage-resistant mutant bacteria [120,121,122,123]. Additionally, phages could be structurally engineered or combined with other antimicrobial compounds, such as antibiotics, to enhance the efficacy of eliminating microbial activity [124]. In future research, the phage-derived enzymes may be studied as biological antibacterial agents to control Enterococcus and its biofilm.

## 5. Conclusions

Phage vB_Efs8_KEN04 is a lytic phage belonging to the genus *Kochikohdavirus* in the family *Herelleviridae*. It was isolated from a municipal sewage treatment plant located in Nairobi East. Phage vB_Efs8_KEN04 exhibits efficacy against all the clinical multidrug-resistant strains of *E. faecalis* tested and one *E. faecium* isolate, including the ability to destroy bacterial biofilms. The genome analysis revealed that the phage lacks genes of concern, including virulence, antibiotic resistance, and lysogeny genes. The phage vB_Efs8_KEN04 has great potential as a candidate for phage therapy against enterococci infections and for controlling biofilms. While this phage shows promise as a therapeutic candidate, addressing safety and regulatory concerns is essential to its successful use in therapy. Continued research, including clinical trials, to demonstrate safety and efficacy in accordance with regulatory standards, and collaboration between regulatory agencies and researchers are crucial for implementing phage therapy responsibly.

## 6. Limitations

A limitation of this study is the lack of animal models as representative models of enterococci infection in humans as the experimental models can mimic the pathogenesis of natural disease.

## Figures and Tables

**Table 1:** Host range analysis of Enterococcus phage vB_Efs8_KEN04.

No.	Bacterial Isolates	Sequence Types (ST)	Origin	Spot Assay	Efficiency of Plating (EOP)
1	*E. faecalis* EFS8 *	1904	Urinary tract infection	++	1
2	*E. faecalis* EFS1	6	Skin and soft tissue infection	++	0.15
3	*E. faecalis* EFS4	947	Skin and soft tissue infection	+	<0.001
4	*E. faecalis* EFS5	6	Skin and soft tissue infection	++	0.076
5	*E. faecalis* EFS6	6	Skin and soft tissue infection	++	0.05
6	*E. faecalis* EFS9	6	Urinary tract infection	++	0.01
7	*E. faecalis* EFS10	6	Urinary tract infection	++	1.1
8	*E. faecalis* EFS11	368	Urinary tract infection	++	0.0004
9	*E. faecalis* EFS13	59	Skin and soft tissue infection	++	0.5
10	*E. faecalis* EFS14	6	Skin and soft tissue infection	++	1.7
11	*E. faecalis* EFS15	16	Urinary tract infection	+	<0.001
12	*E. faecalis* EFS17	6	Skin and soft tissue infection	+	<0.001
13	*E. faecalis* EFS18	368	Urinary tract infection	++	0.0011
14	*E. faecalis* EFS19	1907	Urinary tract infection	++	0.12
15	*E. faecalis* EFS21	44	Skin and soft tissue infection	+	<0.001
16	*E. faecalis* EFS22	1908	Skin and soft tissue infection	+	<0.001
17	*E. faecalis* EFS23	6	Urinary tract infection	++	3
18	*E. faecalis* EFS25	6	Surgical site infection	++	1.5
19	*E. faecalis* EFS26	6	Skin and soft tissue infection	++	1.2
20	*E. faecalis* EFS27	1903	Urinary tract infection	++	0.14
21	*E. faecalis* EFS28	28	Skin and soft tissue infection	++	0.6
22	*E. faecalis* EFS29	6	Blood infection	++	0.8
23	*E. faecalis* EFS30	28	Skin and soft tissue infection	++	0.8
24	*E. faecalis* EFS31	6	Urinary tract infection	++	1.2
25	*E. faecalis* EFS32	1903	Urinary tract infection	++	0.9
26	*E. faecalis* EFS33	1903	Skin and soft tissue infection	++	6
27	*E. faecium* EFM5	80	Urinary tract infection	+	<0.001
28	*E. faecium* EFM1		Skin and soft tissue infection	−	N/A
29	*E. faecium* EFM2	80	Skin and soft tissue infection	−	N/A
30	*E. faecium* EFM3		Skin and soft tissue infection	−	N/A
31	*E. faecium* EFM4	80	Skin and soft tissue infection	−	N/A
32	*E. faecium* EFM6	612	Skin and soft tissue infection	−	N/A
33	*E. faecium* EFM7	761	Skin and soft tissue infection	−	N/A
34	*E. faecium* EFM8	80	Urinary tract infection	−	N/A
35	*E. faecium* EFM9	80	Skin and soft tissue infection	−	N/A
36	*E. faecium* EFM10	2672	Urinary tract infection	−	N/A
37	*E. faecium* EFM11	761	Surgical site infection	−	N/A

EFS, *E. faecalis*; EFM, *E. faecium*; EOP, efficiency of plating. The EOP was determined by dividing the mean plaque-forming units (PFU) of the target bacteria by the mean PFU of the host bacteria (EFS8). * Host bacteria; ++, very clear plaques; +, turbid plaques; −, no plaques; N/A, not applicable.

**Table 2:** Prediction of phage vB_Efs8_KEN04 host range using bioinformatics method.

Protein Name	Phage Name	% Identity	Protein Accession Number	Protein Length	Phage Hosts
Tail fiber protein *	Enterococcus phage vB_Efs8_KEN04	100%	WZP34890.1	1832	*E. faecalis* EFS8
Putative tail fiber protein	Enterococcus phage MDA2	99.89%	QVW28137.1	1825	*E. faecium* VREfm (VRE001, VRE004, VRE008, VRE1147, VRE1181) [73]
Putative tail fiber	Enterococcus phage phiM1EF22	99.95%	BBE37304.1	1822	*E. faecalis* KUEF22 [74]
Tail fiber protein	Enterococcus phage ECP3	99.89%	YP_009147083.1	1822	*E. faecalis* 10K28 [75]
Putative tail fiber	Enterococcus phage phiEF17H	99.89%	BBE37101.1	1822	*E. faecalis* EF17 [74]
Tail fiber protein	Enterococcus phage vB_OCPT_Carl	98.68%	UQT00063.1	1825	*E. faecalis* strains (DP11, EF07, EF116PII, EF11, EF09PII, Ent6, V587, Yi6-1) [76]
Tail fiber protein	Enterococcus phage EF24C	98.47%	YP_001504140.1	1825	*E. faecalis* EF24 [77]
Tail fiber protein	Enterococcus phage vB_OCPT_Bob	98.30%	UQT00475.1	1825	*E. faecalis* strains (B3286, DP6, EF06, DP11, EF07, EF116PII, EF11, EF09PII, Ent6, V587, Yi6-1) [76]
Tail fiber protein	Enterococcus phage vB_OCPT_Car	97.10%	UQT00278.1	1825	*E. faecalis* strains (DP11, EF07, EF116PII, EF11, EF09PII, Ent6, V587, Yi6-1) [76]
Putative tail fiber	Enterococcus phage Sw5	96.38%	USL84310.1	1825	*E. faecalis* OG1RF
Tail fiber protein	Enterococcus phage vB_Efa29212_3e	96.66%	UYB00790.1	1825	*E. faecalis* ATCC 29212TM [78]
Tail fiber protein	Enterococcus phage EFLK1	95.99%	YP_009219864.2	1822	*E. faecalis* V583 and *E. faecalis* V583 phage-resistant mutant (EFDG1r) [79,80]

* The star indicates that the Enterococcus phage vB_Efs8_KEN04 tail fiber protein sequence was used for the Blastp similarity searches.

## Data Availability

Data are contained within the article or Supplementary Materials. The raw data are available at https://www.ncbi.nlm.nih.gov/biosample/, accessed on 25 March 2024, under the bioSample accession number SAMN40604471 and the complete genome of Enterococcus phage vB_Efs8_KEN04 is available at https://www.ncbi.nlm.nih.gov/nuccore/, accessed on 27 April 2024, under the GenBank accession number PP582180.

## Supplementary Materials

The following supporting information can be downloaded at: https://www.mdpi.com/article/10.3390/v16081275/s1, Table S1: Temperature stability test (Mean ± SD); Table S2: pH stability test (Mean ± SD); Table S3: Biofilm formation profile of MDR *Enterococcus faecalis* isolates; Table S4: Biofilm inhibition of phage vB_Efs8_KEN04; Table S5: Biofilm disruption of phage vB_Efs8_KEN04; Table S6: Predicted molecular function for gene products of phage vB_Efs8_KEN04; Table S7: Summary of similar genomic sequence with phage vB_Efs8_KEN04.
