# Supplementary material for: Characterization and Anti-Biofilm Activity of Lytic Enterococcus Phage vB_Efs8_KEN04 against Clinical Isolates of Multidrug-Resistant Enterococcus faecalis in Kenya
Source: Viruses. 2024 Aug 9;16(8):1275. doi: 10.3390/v16081275 (PMC11360260; doi:10.3390/v16081275)
Supplement: Supplementary file 1 [file viruses-16-01275-s001.zip › viruses-3129153-supplementary.pdf]

Table S1. Temperature stability test (Mean ± SD)

Temperature	Log10 (PFU/mL)
-80°C	9.398 ± 0.247
-20°C	9.452 ± 0.255
+4°C	9.222 ± 0.261
20°C	9.501 ± 0.274
22-30°C	9.544 ± 0.274
37°C	9.544 ± 0.254
40°C	8.669 ± 0.165
45°C	8.125 ± 0.127
50°C	8.054 ± 0.145
60°C	7.398

Table S2. pH stability test (Mean ± SD)

pH	Log10 (PFU/mL)
1	0
3	2.176 ± 1.840
5	10.477 ± 1.690
7	12.067 ± 1.883
7.5	12.073 ± 2.095
9	11.875 ± 2.396
11	11.589 ± 2.897
13	0

Table S3. Biofilm formation profile of MDR *Enterococcus faecalis* isolates

No.	Bacterial isolates	Mean OD ± SD	Biofilm formation phenotype
1	ATCC 29212 (Positive Control)	0.881 ± 0.021	strong
2	EFS4 (Negative Control)	0.049 ± 0.001	None biofilm formers (7.69%)
3	*E. faecalis* EFS13	0.048 ± 0.003	
4	*E. faecalis* EFS32	0.102 ± 0.007	Weak (3.85%)
5	*E. faecalis* EFS18	0.187 ± 0.031	Moderate (3.85%)
6	*E. faecalis* EFS8 *	0.427 ± 0.087	Strong (92.31%)
7	*E. faecalis* EFS1	0.445 ± 0.008	
8	*E. faecalis* EFS5	0.407 ± 0.045	
9	*E. faecalis* EFS6	0.364 ± 0.071	
10	*E. faecalis* EFS9	0.496 ± 0.042	
11	*E. faecalis* EFS10	0.463 ± 0.044	
12	*E. faecalis* EFS11	0.282 ± 0.010	
13	*E. faecalis* EFS14	0.370 ± 0.033	
14	*E. faecalis* EFS15	0.387 ± 0.015	
15	*E. faecalis* EFS17	0.36 ± 0.091	
16	*E. faecalis* EFS19	0.452 ± 0.044	
17	*E. faecalis* EFS21	0.31 ± 0.002	
18	*E. faecalis* EFS22	0.423 ± 0.0967	
19	*E. faecalis* EFS23	0.358 ± 0.036	
20	*E. faecalis* EFS25	0.455 ± 0.079	
21	*E. faecalis* EFS26	0.437 ± 0.078	
22	*E. faecalis* EFS27	0.519 ± 0.024	
23	*E. faecalis* EFS28	1.129 ± 0.072	
24	*E. faecalis* EFS29	0.575 ± 0.021	
25	*E. faecalis* EFS30	0.522 ± 0.003	
2	*E. faecalis* EFS31	0.522 ± 0.016	
27	*E. faecalis* EFS33	0.293 ± 0.005	

* Host bacteria

Table S4. Biofilm inhibition of phage vB_Efs8_KEN04

No.	Bacterial isolates	Mean OD ± SD of untreated samples	Mean OD ± SD of treated samples
1	*E. faecalis* EFS8 *	0.310 ± 0.021	0.206 ± 0.023
2	*E. faecalis* EFS1	0.296 ± 0.001	0.179 ± 0.032
3	*E. faecalis* EFS5	0.312 ± 0.011	0.353 ± 0.016
4	*E. faecalis* EFS6	0.302 ± 0.007	0.251 ± 0.036
5	*E. faecalis* EFS9	0.297 ± 0.005	0.202 ± 0.023
6	*E. faecalis* EFS10	0.317 ± 0.029	0.067 ± 0.004
7	*E. faecalis* EFS11	0.196 ± 0.011	0.083 ± 0.018
8	*E. faecalis* EFS14	0.289 ± 0.007	0.163 ± 0.006
9	*E. faecalis* EFS15	0.125 ± 0.006	0.178 ± 0.017
10	*E. faecalis* EFS17	0.285 ± 0.012	0.264 ± 0.013
11	*E. faecalis* EFS18	0.152 ± 0.010	0.004 ± 0.010
12	*E. faecalis* EFS19	0.312 ± 0.009	0.180 ± 0.033
13	*E. faecalis* EFS21	0.321 ± 0.02	0.314 ± 0.018
14	*E. faecalis* EFS22	0.290 ± 0.016	0.282 ± 0.0123
15	*E. faecalis* EFS23	0.325 ± 0.01	0.195 ± 0.012
16	*E. faecalis* EFS25	0.321 ± 0.005	0.201 ± 0.015
17	*E. faecalis* EFS26	0.312 ± 0.019	0.295 ± 0.030
18	*E. faecalis* EFS27	0.226 ± 0.008	0.18 ± 0.015
19	*E. faecalis* EFS28	0.247 ± 0.004	0.144 ± 0.004
20	*E. faecalis* EFS29	0.321 ± 0.017	0.195 ± 0.03
21	*E. faecalis* EFS30	0.259 ± 0.007	0.228 ± 0.009
22	*E. faecalis* EFS31	0.272 ± 0.021	0.206 ± 0.029
23	*E. faecalis* EFS32	0.140 ± 0.002	0.131 ± 0.002
24	*E. faecalis* EFS33	0.158 ± 0.001	0.153 ± 0.002

*Host bacteria

Table S5. Biofilm disruption of phage vB_Efs8_KEN04

No.	Bacterial isolates	Mean OD ± SD of untreated samples	Mean OD ± SD of treated samples
1	*E. faecalis* EFS8 *	0.268 ± 0.012	0.018 ± 0.002
2	*E. faecalis* EFS1	0.298 ± 0.013	0.02 ± 0.009
3	*E. faecalis* EFS5	0.249 ± 0.018	0.004 ± 0.002
4	*E. faecalis* EFS6	0.257 ± 0.008	0.014 ± 0.016
5	*E. faecalis* EFS9	0.256 ± 0.006	0.025 ± 0.002
6	*E. faecalis* EFS10	0.295 ± 0.009	0.019 ± 0.006
7	*E. faecalis* EFS11	0.194 ± 0.025	0.074 ± 0.002
8	*E. faecalis* EFS14	0.319 ± 0.007	0.086 ±0.002
9	*E. faecalis* EFS15	0.129 ± 0.007	0.098 ± 0.008
10	*E. faecalis* EFS17	0.305 ± 0.010	0.171 ± 0.054
11	*E. faecalis* EFS18	0.142 ± 0.003	0.125 ± 0.023
12	*E. faecalis* EFS19	0.382 ± 0.057	0.039 ± 0.013
13	*E. faecalis* EFS21	0.368 ± 0.013	0.078 ± 0.005
14	*E. faecalis* EFS22	0.417 ± 0.017	0.058 ± 0.009
15	*E. faecalis* EFS23	0.6 ± 0.032	0.017 ± 0.007
16	*E. faecalis* EFS25	0.35 ± 0.012	0.029 ± 0.018
17	*E. faecalis* EFS26	0.243 ± 0.027	0.031 ± 0.005
18	*E. faecalis* EFS27	0.178 ± 0.030	0.021 ± 0.010
19	*E. faecalis* EFS28	0.436 ± 0.038	0.016 ± 0.001
20	*E. faecalis* EFS29	0.365 ± 0.006	0.036 ± 0.013
21	*E. faecalis* EFS30	0.37 ± 0.025	0.006 ± 0.001
22	*E. faecalis* EFS31	0.324 ± 0.014	0.031 ± 0.005
23	*E. faecalis* EFS32	0.152 ± 0.006	0.065 ± 0. 012
24	*E. faecalis* EFS33	0.141 ± 0.006	0.023 ± 0.022

* Host bacteria

Table S6. Predicted molecular function for gene products of phage vB_Efs8_KEN04

CDS	Strand	Start (bp)	End (bp)	Length (bp)	Predicted protein function
1	+	134	340	207	hypothetical protein
2	+	417	665	249	hypothetical protein
3	+	743	988	246	hypothetical protein
4	+	1,055	1,285	231	hypothetical protein
5	+	1,336	1,542	207	hypothetical protein
6	-	1,509	1,661	153 bp	hypothetical protein
7	+	1,723	1,893	171	hypothetical protein
8	+	1,973	2,275	303	hypothetical protein
9	+	2,381	2,812	431	hypothetical protein
10	+	2,971	3,183	213	hypothetical protein
11	+	3,180	3,485	306	hypothetical protein
12	+	3,473	3,574	102	hypothetical protein
13	+	3,587	3,946	360	hypothetical protein
14	+	3,951	4,052	102	hypothetical protein
15	-	4,774	4,893	120	hypothetical protein
16	+	4,926	5,165	240	hypothetical protein
17	+	5,272	5,967	696	hypothetical protein
18	+	6,074	6,217	144	hypothetical protein
19	+	6,301	6,567	267	hypothetical protein
20	+	6,640	6,780	141	hypothetical protein
21	+	6,870	7,013	144	hypothetical protein
22	+	7,044	7,133	90	hypothetical protein
23	-	7,289	7,609	321	hypothetical protein
24	-	7,663	7,974	312	membrane protein
25	-	8,013	8,108	96	hypothetical protein
26	-	8,080	8,802	723	hypothetical protein
27	-	8,799	9,068	270	hypothetical protein
28	-	9,069	9,374	306	hypothetical protein
29	-	9,595	9,786	192	hypothetical protein
30	-	9,830	10,054	225	hypothetical protein
31	-	10,068	10,319	252	hypothetical protein
32	-	10,320	10,700	381	hypothetical protein
33	-	10,713	10,910	198	hypothetical protein
34	-	10,910	11,425	516	hypothetical protein
35	-	11,438	11,686	249	hypothetical protein
36	-	11,699	12,223	525	hypothetical protein
37	-	12,224	12,616	393	hypothetical protein
38	-	12,609	12,968	360	hypothetical protein
39	-	12,972	13,142	171	hypothetical protein
40	-	13,130	13,672	543	hypothetical protein
41	-	13,675	14,139	465	hypothetical protein
42	-	14,136	14,606	471	hypothetical protein
43	-	14,622	14,714	93	hypothetical protein
44	-	14,809	15,174	366	hypothetical protein
45	-	15,171	15,350	180	hypothetical protein
46	-	15,364	15,882	519	hypothetical protein
47	-	15,883	16,356	474	hypothetical protein
48	-	16,353	17,063	711	minor head protein
49	-	17,089	17,529	441	replication initiation protein
50	-	17,579	17,767	189	hypothetical protein

51	-	17,764	18,225	462	hypothetical protein
52	-	18,239	18,415	177	hypothetical protein
53	-	18,412	18,609	198	hypothetical protein
54	-	18,606	19,043	438	hypothetical protein
55	-	19,136	19,819	684	phosphoesterase
56	-	19,873	20,352	480	hypothetical protein
57	-	20,356	20,607	252	hypothetical protein
58	-	20,604	21,215	612	phosphoesterase
59	-	21,226	21,543	318	membrane protein
60	-	21,573	21,695	123	hypothetical protein
61	-	21,776	22,189	414	hypothetical protein
62	-	22,213	23,073	861	hypothetical protein
63	-	23,166	23,339	174	hypothetical protein
64	-	23,329	23,673	345	hypothetical protein
65	-	23,673	23,927	255	hypothetical protein
66	-	23,908	24,162	255	hypothetical protein
67	-	24,173	24,508	336	hypothetical protein
68	-	24,483	24,920	438	hypothetical protein
69	-	24,936	25,397	462	hypothetical protein
70	-	25,390	25,617	228	hypothetical protein
71	-	25,605	26,036	432	hypothetical protein
72	-	26,039	26,269	231	hypothetical protein
73	-	26,266	26,697	432	hypothetical protein
74	-	26,701	26,853	153	hypothetical protein
75	-	26,853	27,800	948	thymidylate synthase
76	-	27,850	28,236	387	hypothetical protein
77	-	28,329	28,478	150	hypothetical protein
78	-	28,478	29,335	858	lipoprotein
79	-	29,497	30,480	984	ribonucleotide reductase class Ia beta subunit
80	-	30,493	32,643	2,151	ribonucleotide reductase large subunit
81	-	32,646	32,888	243	thioredoxin domain
82	-	33,016	33,222	207	hypothetical protein
83	-	33,288	33,593	306	hypothetical protein
84	-	33,684	33,914	231	transcriptional regulator
85	-	33,978	34,202	225	hypothetical protein
86	-	34,312	34,596	285	hypothetical protein
87	-	34,745	35,836	1,092	ATPase
88	-	35,903	36,058	156	hypothetical protein
89	-	36,186	36,368	183	hypothetical protein
90	-	36,368	36,475	108	hypothetical protein
91	-	36,726	36,872	147	hypothetical protein
92	+	36,846	36,992	147	hypothetical protein
93	+	37,048	37,170	123	hypothetical protein
94	-	37,728	37,823	96	hypothetical protein
95	+	38,055	38,177	123	hypothetical protein
96	+	38,267	38,359	93	hypothetical protein
97	+	38,655	38,756	102	hypothetical protein
98	+	38,713	38,835	123	hypothetical protein
99	-	38,986	39,120	135	hypothetical protein
100	+	39,151	39,306	156	hypothetical protein
101	+	39,359	39,454	96	hypothetical protein
102	+	39,454	39,552	99	hypothetical protein
103	+	39,591	39,683	93	hypothetical protein

104	-	39,799	40,137	339	virion structural protein
105	-	40,201	40,545	345	hemolysin
106	-	40,580	40,840	261	membrane protein
107	+	40,957	41,226	270	membrane protein
108	+	41,247	41,525	279	hypothetical protein
109	+	41,529	41,957	429	terminase small subunit
110	+	41,957	42,160	204	terminase large subunit
111	-	42,119	42,226	108	hypothetical protein
112	-	42,340	42,453	114	hypothetical protein
113	+	42,416	43,381	996	homing endonuclease
114	+	43,540	44,325	786	terminase large subunit
115	-	44,307	44,456	150	hypothetical protein
116	+	44,573	45,511	939	HNH endonuclease
117	+	45,624	46,388	765	terminase large subunit
118	+	46,488	47,276	789	virion structural protein
119	+	47,381	48,097	717	hypothetical protein
120	+	48,087	48,431	345	hypothetical protein
121	+	48,525	49,394	870	endolysin
122	+	49,561	50,199	639	transglycosylase
123	+	50,342	50,686	345	portal protein
124	-	50,772	50,867	96	hypothetical protein
125	+	50,896	52,425	1,530	portal protein
126	+	52,432	52,545	114	hypothetical protein
127	+	52,517	53,323	807	head maturation protease
128	+	53,330	54,259	930	hypothetical protein
129	+	54,400	55,794	1,395	major head protein
130	+	55,899	56,162	264	hypothetical protein
131	+	56,175	57,074	900	tail fiber protein
132	+	57,094	57,963	870	virion structural protein
133	+	57,956	58,579	624	hypothetical protein
134	+	58,583	59,428	846	hypothetical protein
135	+	59,428	59,661	234	hypothetical protein
136	+	59,665	61,374	1,710	tail sheath
137	+	61,435	61,857	423	virion structural protein
138	+	61,947	62,093	147	hypothetical protein
139	+	62,230	62,703	474	tail assembly chaperone
140	+	62,771	63,346	576	RNA polymerase beta subunit
141	+	63,391	67,047	3,657	tail associated lysin
142	+	67,086	70,271	3,186	tail protein with lysin activity
143	+	70,335	75,833	5,499	tail fiber protein
144	+	75,933	78,344	2,412	tail protein
145	+	78,338	79,063	726	tail fiber protein
146	+	79,095	79,241	147	hypothetical protein
147	+	79,316	79,411	96	hypothetical protein
148	+	79,378	80,067	690	virion structural protein
149	+	80,071	80,607	537	hypothetical protein
150	+	80,594	81,298	705	baseplate protein
151	+	81,314	82,366	1,053	baseplate wedge subunit
152	+	82,384	84,795	2,412	hypothetical protein
153	-	84,810	84,920	111	hypothetical protein
154	+	84,967	85,446	480	virion structural protein
155	+	85,461	88,925	3,465	hypothetical protein
156	+	89,006	89,968	963	HNH endonuclease

157	+	90,047	90,253	207	hypothetical protein
158	+	90,499	92,271	1,773	DNA helicase
159	+	92,299	93,927	1,629	HTH DNA binding protein
160	+	93,960	96,494	2,535	DarB-like antirestriction
161	+	96,494	97,549	1,056	SbcD-like subunit of palindrome specific endonuclease
162	+	97,665	99,557	1,893	exonuclease
163	+	99,566	100,231	666	anti-sigma factor
164	+	100,232	101,290	1,059	DNA primase
165	+	101,310	101,942	633	hypothetical protein
166	+	101,971	102,855	885	dUTPase
167	+	102,858	103,088	231	dUTPase
168	+	103,090	103,398	309	hypothetical protein
169	+	103,385	103,696	312	hypothetical protein
170	+	103,689	104,060	372	hypothetical protein
171	+	104,080	104,748	669	RusA-like Holliday junction resolvase
172	+	104,750	105,049	300	hypothetical protein
173	+	105,055	105,534	480	hypothetical protein
174	+	105,627	106,421	795	hypothetical protein
175	+	106,414	106,725	312	DNA binding protein
176	+	106,815	109,217	2,403	DNA polymerase
177	-	109,356	109,454	99	hypothetical protein
178	-	109,657	109,746	90	hypothetical protein
179	-	109,767	109,865	99	hypothetical protein
180	+	109,846	110,325	480	DNA polymerase
181	+	110,429	110,971	543	hypothetical protein
182	+	111,027	112,313	1,287	single strand DNA binding protein
183	+	112,398	112,937	540	UvsX-like recombinase
184	-	112,995	113,102	108	hypothetical protein
185	-	113,164	113,265	102	hypothetical protein
186	+	113,248	113,946	699	UvsX-like recombinase
187	+	114,072	114,386	315	hypothetical protein
188	+	114,379	114,993	615	RNA polymerase sigma factor
189	+	115,054	115,329	276	holin
190	+	115,377	116,339	963	structural protein with Ig domain
191	+	116,360	116,803	444	Ig domain containing protein
192	+	116,910	117,215	306	hypothetical protein
193	+	117,212	118,165	954	exopolyphosphatase
194	+	118,219	119,502	1,284	exonuclease
195	+	119,514	119,888	375	hypothetical protein
196	+	119,927	120,547	621	hypothetical protein
197	+	120,547	121,287	741	hypothetical protein
198	+	121,277	121,783	507	hypothetical protein
199	+	121,797	122,147	351	hypothetical protein
200	+	122,172	123,029	858	hypothetical protein
201	+	123,133	123,978	846	hypothetical protein
202	+	123,971	125,599	1,629	hypothetical protein
203	+	125,890	126,030	141	hypothetical protein
204	+	125,918	126,607	690	hypothetical protein
205	+	126,618	127,085	468	virion structural protein
206	+	127,182	129,464	2,283	hypothetical protein
207	+	129,554	129,730	177	hypothetical protein
208	+	129,751	130,257	507	hypothetical protein
209	+	130,332	130,502	171	hypothetical protein

210	+	130,492	130,764	273	hypothetical protein
211	+	130,872	131,123	252	transcriptional repressor
212	+	131,135	131,422	288	hypothetical protein
213	+	131,425	132,213	789	hypothetical protein
214	+	132,295	133,368	1,074	hypothetical protein
215	+	133,499	133,801	303	hypothetical protein
216	+	133,803	134,105	303	hypothetical protein
217	+	134,108	134,401	294	hypothetical protein
218	+	134,398	134,577	180	hypothetical protein
219	+	134,590	134,955	366	hypothetical protein
220	+	134,982	135,359	378	hypothetical protein
221	+	135,352	135,582	231	hypothetical protein
222	+	135,586	135,960	375	hypothetical protein
223	+	135,957	136,214	258	hypothetical protein
224	+	136,253	136,813	561	hypothetical protein
225	+	136,855	137,751	897	hypothetical protein
226	+	137,752	138,381	630	hypothetical protein
227	+	138,459	138,701	243	hypothetical protein
228	+	138,716	138,859	144	hypothetical protein
229	+	138,872	139,066	195	hypothetical protein
230	+	139,082	139,723	642	hypothetical protein
231	+	139,735	140,178	144	hypothetical protein
232	+	140,329	140,760	432	hypothetical protein
233	-	140,902	141,033	132	hypothetical protein
234	-	141,017	141,112	96	hypothetical protein
235	+	141,105	141,404	300	hypothetical protein
236	+	141,476	141,805	330	hypothetical protein
237	+	141,901	142,041	141	hypothetical protein
238	+	142,100	142,327	228	hypothetical protein

Table S7. Summary of similar genomic sequence with phage vB_Efs8_KEN04

No.	Phage name	Query cover	identity	Accession number	Genome size (bp)
	Enterococcus phage vB_Efs8_KEN04			PP582180	142402
1	Enterococcus phage phiM1EF22	94%	99.29%	AP018715.1	143046
2	Enterococcus phage ECP3	96%	99.23%	NC_027335.2	145519
3	Enterococcus phage PBEF129	95%	99.02%	MN854830.2	141520
4	Enterococcus phage vB_EfaM_Ef2.3	95%	99.02%	MK721192.1	147289
5	Enterococcus phage Sw5	94%	98.47%	ON286976.1	143759
6	Enterococcus phage vB_EfaM_Ef2.1	92%	97.70%	MK693030.1	140938
7	Enterococcus phage 53	91%	97.00%	OQ420427.1	139913
8	Enterococcus phage EFLK1	85%	97.91%	NC_029026.1	130952
9	Enterococcus phage vB_EfaH_EF1TV	92%	97.71%	MK268686.1	143507
10	Enterococcus phage vB_OCPT_Carl	90%	98.01%	ON113167.1	143879
11	Enterococcus phage vB_OCPT_Car	89%	97.55%	ON113168.1	141483
12	Enterococcus phage phiEF17H	93%	98.37%	AP018714.1	143638
13	Enterococcus phage phi EF19G	93%	98.37%	LC596379.1	143400
14	Enterococcus phage phi EF7H	93%	98.37%	LC596377.1	143399
15	Enterococcus phage phi EF14H1	93%	98.36%	LC596378.1	143280
16	Enterococcus phage MDA2	93%	97.62%	MW633168.1	140226
17	Enterococcus phage EF24C	92%	97.28%	NC_009904.1	142072
18	Enterococcus phage phiEF24C-P2	92%	97.28%	NC_047853.1	142072
19	Enterococcus phage 156	87%	97.27%	LR031359.1	141133
20	Enterococcus phage vB_OCPT_Bob	92%	97.07%	ON113169.1	142921
21	Enterococcus phage EFGrKN	18%	83.04%	MW004544.1	147532
22	Enterococcus phage EFDG1	18%	82.89%	NC_029009.1	147589
23	Enterococcus phage phiSHEF13	23%	78.30%	OL799258.1	151389
24	Enterococcus phage vB_OCPT_Ben	18%	78.19%	MN027503.1	151985
25	Enterococcus phage phiSHEF16	21%	78.17%	OL799260.1	151935
26	Enterococcus phage GVEsP-1	18%	78.17%	MZ333462.1	149913
27	Enterococcus phage EfV12-phi1	21%	78.15%	NC_048087.1	152770
28	Enterococcus phage EFGrNG	18%	78.13%	MW004545.1	145199
29	Enterococcus phage vB_OCPT_Bill	18%	78.19%	OM966901.1	147049
30	Enterococcus phage vB_OCPT_CCS1	21%	78.11%	ON113170.1	150888
31	Enterococcus phage vB_OCPT_Bop	19%	78.11%	ON125307.1	153454
32	Enterococcus phage UTI-EfS3	18%	78.11%	OL870611.1	150393
33	Enterococcus phage vB_EfaM_A2	18%	78.10%	MT856905.1	149431
34	Enterococcus phage PEf771	18%	78.10%	MN241318.1	151052
35	Enterococcus phage Porthos	20%	78.08%	LR990835.2	151703
36	Enterococcus phage EFP01	22%	78.06%	NC_047796.1	155053
37	Enterococcus phage iF6	20%	78.14%	MT909815.1	156592
